# Enhancing Fatigue Performance of Coal Gangue Concrete (CGC) through Polypropylene Fiber Modification: Experimental Evaluation

**DOI:** 10.3390/polym16081096

**Published:** 2024-04-15

**Authors:** Di Wu, Laiwang Jing, Yan Li, Tao Ran, Shaochi Peng, Wei Jing

**Affiliations:** 1 School of Civil Engineering and Architecture, Anhui University of Science and Technology, Huainan 232000, China; 2 School of Computing, Macquarie University, Sydney 2109, Australia; 3 School of Environment and Civil Engineering, Chengdu University of Technology, Chengdu 610000, China

**Keywords:** polypropylene fibers, coal gangue, fiber-reinforced concrete, cyclic loading and unloading, energy evolution, SEM test

## Abstract

Coal gangue is a byproduct of coal mining and processing, and according to incomplete statistics, China has amassed a substantial coal gangue stockpile exceeding 2600 large mountains, which poses a serious threat to the ecological environment. Utilizing gangue as a coarse aggregate to produce gangue concrete (GC) presents a promising avenue for addressing the disposal of coal gangue; however, gangue concrete presents several challenges that need to be tackled, such as low strength and poor resistance to repeated loads. In this study, polypropylene fibers (PPFs) were incorporated into gangue concrete to enhance its utilization rate. Uniaxial compressive and repeated loading experiments were then conducted to investigate the uniaxial strength and fatigue properties of polypropylene fiber-reinforced gangue concrete (PGC) with varying gangue substitution rates (20%, 40%, and 60%) and different polypropylene fiber admixtures (0, 0.1%, 0.2%, and 0.3%). The findings indicate that incorporating gangue at a substitution rate of 40% could notably enhance the uniaxial compressive strength of PGC, resulting in a maximum increase of 19.4%. In the repeated loading experiments, the ductility of PGC was enhanced with the incorporation of PPFs, resulting in a reduction of 33.76% in the damage factor and 19.42% in residual strain for PGC-40-0.2 compared to PGC-40-0. A PPF content of 0.2% was found to be optimal for enhancing the fatigue performance of PGC. Scanning electron microscope (SEM) testing proved the improvement effect of polypropylene fiber on gangue concrete from a microscopic perspective. This study provides crucial experimental data and a theoretical foundation for the utilization of gangue concrete in complex stress environments.

## 1. Introduction

Coal holds paramount significance in China as one of the primary sources of energy [1]. As the world’s foremost producer and consumer of coal, China has depended on this resource to underpin its rapid economic expansion [2]. Coal finds extensive utilization in power generation, industrial production, construction, and various other sectors, furnishing a stable energy foundation for the nation. Simultaneously, the coal industry generates a substantial number of employment opportunities, contributing to the maintenance of social stability [3].

Coal gangue constitutes a byproduct generated during coal mining and handling processes, encompassing coal, rock, and other impurities [4]. The treatment of coal gangue is imperative for environmental protection, resource utilization, and fostering sustainable development.

Primarily, the treatment of coal gangue serves to mitigate environmental pollution [5,6]. Coal gangue encompasses substantial quantities of deleterious substances, including heavy metals and sulfides [7], and if discharged into the environment without effective treatment, it has the potential to pollute soil, water bodies, and the atmosphere [8]. Such pollution not only jeopardizes the health of the ecosystem but also exerts a detrimental impact on the well-being of the surrounding population [9]. By employing scientifically and rationally designed coal gangue treatment technologies, the release of harmful substances can be minimized, thereby reducing the risk of environmental pollution. Secondly, coal gangue treatment facilitates the efficient utilization of resources [10]. Gangue harbors underexploited coal, valuable minerals, and other recoverable resources [11]. Through the application of advanced gangue sorting technology, these valuable components can be separated and utilized in various domains, including energy production, building material preparation, and industrial raw material extraction. This not only enhances the efficiency of resource utilization but also alleviates development pressures on the pristine environment. Thirdly, the treatment of coal gangue contributes to the enhancement of the ecological environment in the vicinity of coal mines. The gangue area typically represents barren and unproductive land, unsuitable for normal agricultural or vegetative growth [9]. Through scientific and rational gangue management, it is possible to restore and improve these damaged lands [12]. The aim is to enhance the ecological quality of the surrounding area by reinstating vegetation and conducting soil remediation, rendering abandoned land ecologically functional once more [13]. Furthermore, the proper handling of gangue contributes to the mitigation of geological hazards. The large-scale accumulation of coal gangue not only elevates the risk of geological disasters, such as landslides and mudslides, but may also result in issues such as groundwater pollution and land subsidence. Through the judicious treatment of gangue, the likelihood of these geological disasters can be diminished, thereby enhancing the overall safety of the area. The published statistics indicate that China has amassed a substantial coal gangue stockpile, exceeding 2600 large mountains, with a cumulative total exceeding 6 billion tonnes. Moreover, the annual accumulation persists at a rate exceeding 300 million tonnes. The disposal of coal gangue is therefore an urgent issue.

In pursuit of gangue reuse, numerous scholars are currently making substantial research efforts. Deluan [14] conducted research on the preparation of alkali-activated geopolymer materials utilizing gangue and by-products from municipal waste incineration; Yu [15] extracted aluminum oxide from gangue through the process of hydrochloric acid leaching; Yongda [16] utilized gangue as a sintering aid in the low-temperature sintering process of SiC ceramic films, among other materials.

To achieve the large-scale utilization of gangue, employing it as a substitute for natural gravel in concrete production proves to be an effective approach for resourceful gangue utilization. This not only addresses the myriad issues stemming from gangue accumulation but also compensates for the severe shortage of natural sand and gravel. To date, numerous scholars have undertaken research on gangue-based concrete. Junbo [17] employed gangue as a shotcrete aggregate and utilized machine learning techniques to predict its mechanical properties; Tong [18] conducted experimental and numerical investigations on the mechanical and environmental properties of veinstone fine aggregate concrete and reinforced veinstone fine aggregate concrete, utilizing gangue sand as a fine aggregate with varying veinstone fine aggregate replacement rates; Wen [19] employed gangue as both coarse and fine aggregates in concrete placement and measured the shrinkage of gangue aggregate concrete over a 126-day period. These studies validated the feasibility of utilizing gangue in concrete. Xin [20] developed a lightweight foam concrete using gangue for confinement filling in coal mines. Bo [21] conducted a study on the mechanical properties and size effects of mesoscale coal gangue concrete (CGC). Jisheng [22] investigated the damage constitutive model of CGC under the influence of freeze–thaw cycles. These studies provided evidence supporting the effectiveness of CGC.

While CGC finds widespread application as a green building material for roads, buildings, slope protection works, industrial flooring, pipelines, and tunnels [14,23,24], it is generally not employed in load-bearing areas. The rationale behind this lies in the fact that such scenarios often entail regular reciprocating load disturbances. Due to its inherent water absorption, high porosity, and low strength, CGC often exhibits inferior mechanical properties compared to traditional concrete [25,26,27,28,29,30]. The deficiency in the mechanical properties of CGC has emerged as a significant obstacle to the widespread promotion and application of this material.

The deficiencies in CGC can be effectively mitigated by incorporating fibers into the mixture. Scholars worldwide have extensively investigated this aspect. Bin [31] conducted a study on the reinforcement of gangue aggregate concrete using steel fibers, examining its flexural properties to enable the application of CGC in structural beams. Haochen [32] utilizes corn stover fiber to modify cemented gangue, enabling its application in coal mine backfill areas. Mengyu [33] employed basalt fiber to modify CGC and demonstrated that a CGC mixture with a 0.15% basalt fiber content exhibited optimal strength. Ni’s [34] utilization of glass fiber-reinforced polymers demonstrated their capacity to substitute ordinary concrete without compromising load-bearing capacity. Hongbo [35] modified autogenous gangue aggregate concrete with carbon fiber-reinforced polymers to mitigate their adverse impact on the mechanical properties of concrete. Shengyong [36] improved the axial compressive properties of tubular gangue steel fiber short concrete columns by incorporating glass fiber-reinforced polymer. These experiments thoroughly showcased the feasibility of using fibers for the modification of CGC. After experimentally comparing common fibers available in the market, such as polypropylene fiber, aramid fiber, basalt fiber, glass fiber, carbon fiber, etc., polypropylene fiber, which exhibited the best overall performance, was chosen as a material for modifying CGC in this research.

Polypropylene fiber is a synthetic material derived from polypropylene polymer. Its escalating use in diverse fields is attributed to its unique properties, rendering it an ideal fiber material. Its primary characteristics encompass light weight and high strength, chemical resistance, low moisture absorption, electrical insulation, flame retardancy, low processing cost, recyclability, and environmental friendliness. These attributes have led to its widespread use across various fields, ranging from textiles to engineering materials. research on polypropylene fiber-modified concrete is well established, with numerous scholars making substantial contributions to this field. The incorporation of steel and polypropylene fibers to reinforce lightweight geopolymer concrete, as demonstrated by Ali [37], resulted in enhanced mechanical properties of the concrete; Ninghui [38] reinforced concrete with basalt-polypropylene fibers and examined the cracking resistance mechanism using SEM; Qinghe [39] modified barite aggregate radiation-shielded concrete with polypropylene fibers to enhance its mechanical properties; Hua [40] reinforced coral aggregate concrete with polypropylene fibers and studied their impact on the concrete’s performance.

There are many scholars who have also considered the combined use of gangue and polypropylene fiber, in view of the above characteristics of polypropylene fiber compensating for the defects and insufficiency of gangue mechanical properties: Pang [41] utilized polypropylene fibers mixed with gangue, slag, and fly ash in a highly alkaline environment, in different conditions, carried out unconfined compressive strength testing and three-point bending testing, and performed the detection of its mechanical properties. Yang [42] made polypropylene fiber strips by cutting disposable masks into strips after sterilization and combined them with gangue concrete to observe the changes in its mechanical properties. However, what kind of mechanical modification ability the polypropylene fiber in gangue concrete will show in the face of repeated loading experiments has to be further studied. Yang [43] used gangue to replace the coarse aggregate in ordinary concrete, based on gangue concrete, to study the effect of different volumes of polypropylene fiber on its mechanical properties. The concrete achieved the best mechanical properties when the gangue concrete was modified by using single-mixed fly ash and double-mixed fly ash and mineral powder.

In this study, CGC is prepared by substituting varying volume fractions of coarse aggregate with gangue, and different dosages of polypropylene fiber are introduced to the CGC. This research compares the differences between CGC with different fiber contents in uniaxial compression tests and constant-stress lower limit cyclic loading and unloading experiments. Through these tests, this study analyzes the effects of fiber dosage and gangue content on the dissipation energy and damage factor of cyclic loading experiments of CGC, delving into the intrinsic mechanisms of these influences. This investigation extends to scrutinizing the impact of fiber admixture and gangue content on the dissipation energy and damage factor of CGC cyclic loading experiments and their inherent mechanisms. The outcomes of this study offer insights that can serve as a reference for subsequent research and the practical application of polypropylene fiber CGC in production.

## 2. Materials and Methods

### 2.1. Raw Materials

**Figure 1:**
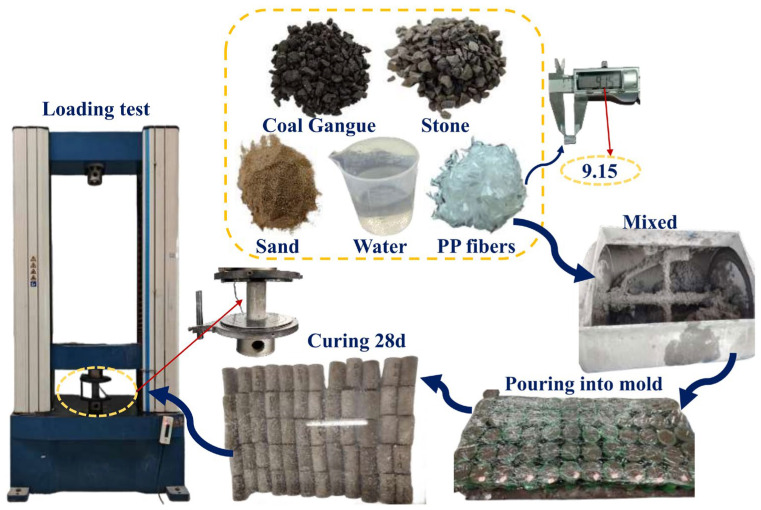
Concrete preparation process.

Bagongshan brand ordinary silicate cement (P.O 42.5) served as the cementitious material in the study. The coarse aggregate used was ordinary gravel with a particle size range of 5–20 mm and an apparent density of 2864 kg/m^3^. The fine aggregate utilized is natural river sand with a fineness modulus of 2.6. Gangue particles were sourced from Xinzhuangzi Coal Mine of Huainan City, as illustrated in Figure 1, possessing a particle size of 5–20 mm, an apparent density of 2600 kg/m^3^, a crushing value of 19.6%, and a water absorption rate of 5.2%. The manufacturer of the polypropylene fiber is China’s Hebei Chuangsheng Chemical Company. The apparent density is 0.91 g/cubic centimeter and the tensile strength is 440 MPa. Ordinary water was used in the experiment.

### 2.2. Mix Design

Following numerous laboratory tests and in accordance with the Ordinary Concrete Proportion Design Regulations (JGJ 55-2011) [44], the water/cement ratio for the concrete was ultimately established at 0.4. The replacement of coarse aggregate (ordinary gravel) in concrete with an equivalent volume of gangue defines the gangue replacement ratio. In this study, the gangue substitution rate was established at three levels: 20%, 40%, and 60%.

At the same gangue substitution rate, various concentrations of polypropylene fibers were implemented, including concentrations of 0%, 0.1%, 0.2%, and 0.3%. The experiment comprised a total of 12 groups of specimens, and the specific mixing ratios are detailed in Table 1. To minimize errors, six samples were utilized in each group. Each sample took on a cylindrical shape with dimensions of Φ 50 mm × 100 mm.

### 2.3. Preparation of Specimens

Due to the high water absorption of gangue, a pre-soaking process in water was conducted to ensure the stability of the water/cement ratio in the concrete before the experiment. At the commencement of the experiment, any water on the surface of the gangue was carefully wiped dry and removed, and set aside. To obtain high-quality concrete samples, specific steps must be followed in the preparation process. Firstly, add the weighed cement, crushed stone, gangue, and sand to the horizontal concrete mixer, and mix thoroughly for 3 min. Continue by adding polypropylene fiber and mixing for an additional 2 min. Subsequently, add the weighed water and continue mixing with the dry ingredients for 3 min to ensure thorough blending of all components. Pour the mixture into the cylindrical molds, shake it well, and smooth the surface with a spatula. Cover the molds with cling film to prevent water evaporation. After a 24 h waiting period, the cured concrete was removed from the molds and transferred to a constant temperature and humidity curing area for a duration of 28 days. Figure 1 illustrates the steps involved in the concrete production process.

### 2.4. Testing Methods

**Figure 2:**
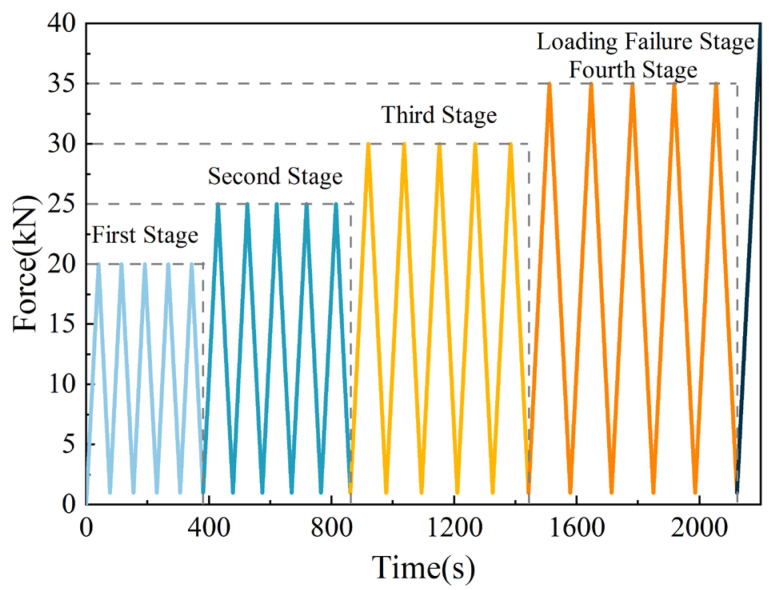
Stress loading path.

Uniaxial compressive and repetitive loading experiments were conducted on the specimens utilizing the RDL-200 electronic creep meter. In the uniaxial compressive test, the loading rate was set at 500 N/s until the specimen was destroyed, at which point the loading was stopped to obtain the peak compressive strength of the specimen. In the repeated load experiments, a preload of 500 N was applied to all specimens to ensure close contact between the specimen surfaces and the apparatus. The loading method involved unequal amplitude loading, with the upper loading limit set at four stages of 20 KN, 25 KN, 30 KN, and 35 KN, respectively. For each stage, the lower cyclic stress limit was maintained at a constant 1 kN to prevent sample slippage. Each stress stage is cycled five times and the last stress stage is cycled, and the stress continues to rise until the specimen is destroyed. The stress loading path is shown in Figure 2.

## 3. Results and Discussion

### 3.1. Uniaxial Compression Experiments

**Figure 3:**
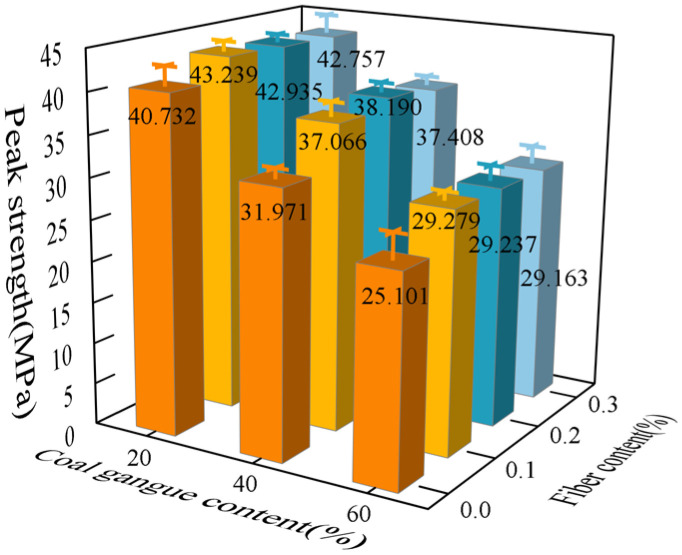
Gangue concrete compressive strength.

The loading rate was maintained at 500 N/s, and the uniaxial compressive strength of the specimen obtained is depicted in Figure 3. The uniaxial compression tests revealed an inverse relationship between the gangue content and the ultimate compressive strength of the specimen, indicating that higher gangue content led to lower ultimate compressive strength. This phenomenon can be attributed to various factors, including the lower strength of the gangue, non-uniform particle distribution, weaker bond with cement, the presence of internal impurities, and increased porosity when compared to ordinary coarse aggregate crushed stone.

For samples with different fiber contents, an increase in strength was observed, with the most obvious strength increase at 0.2% fiber content, with a maximum increase of 19.4%. This is mainly attributed to the tensile properties of concrete reinforced by polypropylene fibers. The main damage mode of concrete under uniaxial compressive load is through crushing and crack expansion. When bearing a load, fibers can inhibit the growth and development of cracks, thereby achieving the effect of enhancing the uniaxial compressive strength of concrete. Despite this, the strength of polypropylene fiber is lower than that of the concrete body, so in gangue fiber concrete, the gangue content is the decisive factor that determines the uniaxial compressive limit of the specimen.

### 3.2. Repeated Loading Experiments

**Figure 4:**
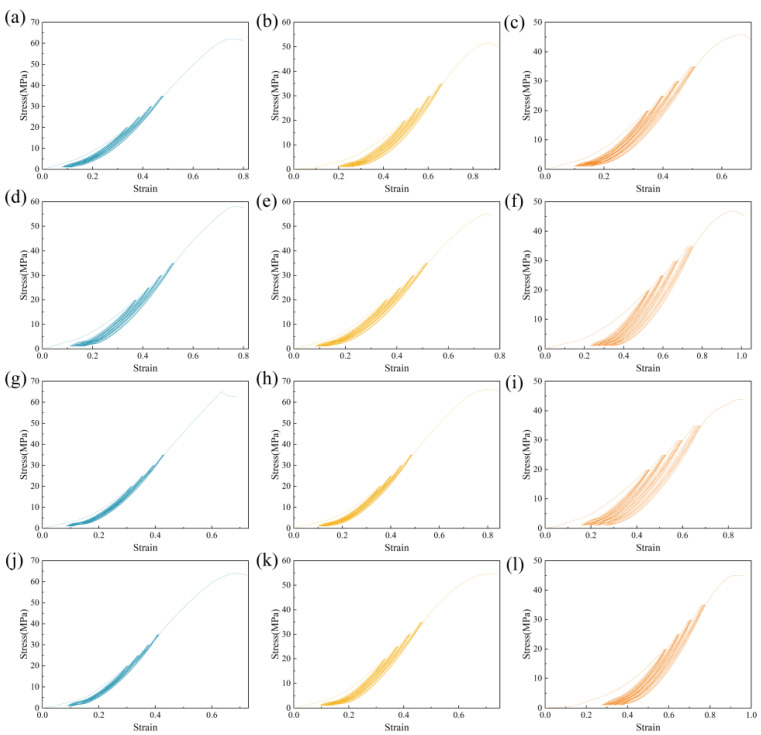
Repeated loading experimental stress–strain curves of gangue fiber concrete. (**a**) GC1; (**b**) GC5; (**c**) GC9; (**d**) GC2; (**e**) GC6; (**f**) GC10; (**g**) GC3; (**h**) GC7; (**i**) GC11; (**j**) GC4; (**k**) GC8; (**l**) GC12.

**Figure 5:**
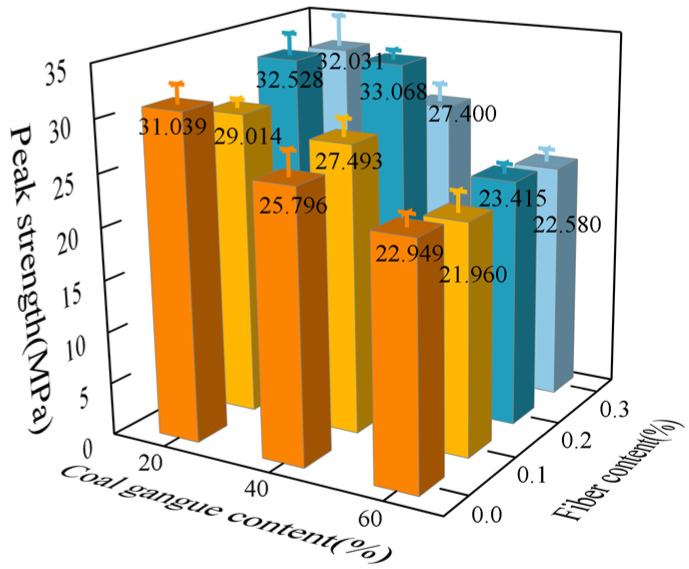
Peak strength of gangue fiber concrete after repeated loading.

After the repeated loading experiments, the ultimate compressive strength of gangue concrete specimens showed a notable increase in all groups with the addition of polypropylene fibers compared to the control group without fibers. The stress–strain curve of the gangue fiber concrete repeated loading experiment is shown in Figure 4. The peak strength of the gangue fiber concrete after repeated loading is shown in Figure 5. The ultimate compressive strength of the samples after cyclic loading damage was notably increased with the addition of polypropylene fibers when the gangue substitution fractions were 20% and 40%. The ultimate compressive strengths of the samples after cyclic loading damage exhibited significant differences under varying conditions of fiber content, particularly when the polypropylene fiber content was 0.2%. In this scenario, the ultimate compressive strength of the samples after cyclic loading damage increased most significantly. However, with a gangue substitution fraction of 60%, the addition of fibers resulted in a smaller increase in the ultimate compressive strength of the samples after cyclic loading damage. The difference between different fiber contents gradually decreased; at a polypropylene fiber content of 0.2%, the ultimate compressive strength of the samples after cyclic loading damage remained the most significantly improved group.

The inclusion of polypropylene fibers can contribute to pore filling, stress dispersion, deformation suppression, and energy consumption during the hardening and loading phases of the specimen. The fibers are distributed across the cracks, which transfers the originally concentrated tensile stress at the crack tips to the interface between the fibers and the substrate, thereby reducing stress concentration at the crack tip. From a deformation perspective, the fibers spanning the crack and anchored in the matrix on both sides effectively restrain the relative expansive deformation of the matrix by utilizing their tensile capacity. The continuous expansion of cracks requires overcoming the bond between the fibers and the matrix, leading to fiber pull-out or even breakage, a process that consumes energy and significantly enhances the ability of gangue concrete to resist repetitive loads. When the polypropylene fiber content reaches 0.3%, instead of increasing, the ultimate strength tends to decrease instead. This result indicates that when the fiber admixture is too large, the crack-blocking effect of polypropylene fibers weakens. The extensive distribution of fibers may lead to clustering, forming cavities in the gangue concrete, ultimately reducing its ultimate strength.

### 3.3. Destroy Form

**Figure 6:**
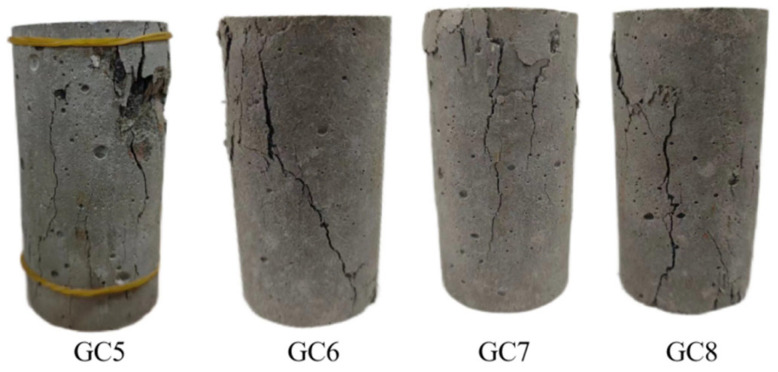
Failure patterns of each group of specimens with 40% coal gangue content.

Figure 6 illustrates the apparent morphology of the sample group containing 40% gangue content after undergoing 20 cycles of repeated loading and reaching the peak strength. This observation aims to investigate the influence of varying fiber contents on the damage morphology of gangue concrete. After 20 cycles of loading, each sample group exhibited varying degrees of deformation. The most pronounced deformation was observed in the GC5 group. Extensive cracks and fragments appeared on the surface of the specimens, displaying a damage pattern characteristic of typical brittle fracture. The length and width of the cracks were greater in comparison to the experimental group with added polypropylene fibers. This damage pattern suggests that the concrete specimens exhibit a deficiency in toughness and tensile strength when subjected to external loading, rendering them susceptible to extensive damage. Upon observing each experimental group with added polypropylene fibers, only minor cracks appeared, predominantly distributed vertically. The apparent morphology of the test blocks remained relatively intact, with no visible shedding of cement. As depicted in the figure, the most severe damage was observed in group GC5, followed by GC6 and GC8. Notably, in specimen GC8, some fine fiber aggregates were observed on the surface of the specimen. These aggregates may result in localized stress concentration, thereby diminishing the overall performance of the specimen. Therefore, despite the addition of more fibers, the damage morphology of the specimen did not exhibit further improvement. The most favorable damage morphology was observed in the GC7 specimen. The cracks on the surface of the specimens were markedly reduced, and their extension was suppressed to a greater extent. The specimens exhibited a certain degree of toughness during the destruction process, displaying relatively flat surfaces and overall high tensile strength.

### 3.4. Energy Evolution

**Figure 7:**
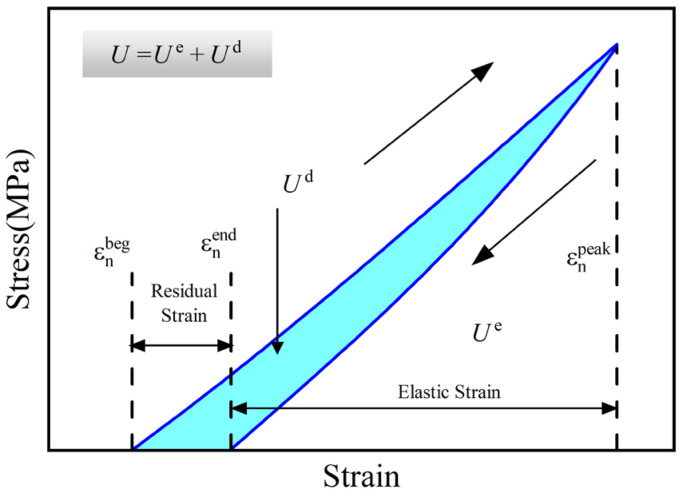
Schematic diagram of energy partitioning.

During cyclic loading, the loading and unloading curves diverge to form hysteresis loops. In a stress–strain diagram, the area below the loading curve represents the mechanical energy density, while the area below the unloading curve represents the elastic energy density. The difference between these two areas indicates energy dissipation. Please refer to Figure 7 for an energy diagram.

The formula for calculating energy density is presented below.

$$\begin{array}{l} U=U^{e}+U^{d}\\ U^{d}=\int \sigma^{up}d\varepsilon^{up}-\int \sigma^{down}d\varepsilon^{down} \end{array}$$

U is the total mechanical energy density, $\mathrm{MJ}/m^{3}$; $U^{e}$ is the elastic energy density, $\mathrm{MJ}/m^{3}$; and $U^{d}$ is the dissipative energy density, $\mathrm{MJ}/m^{3}$.

**Figure 8:**
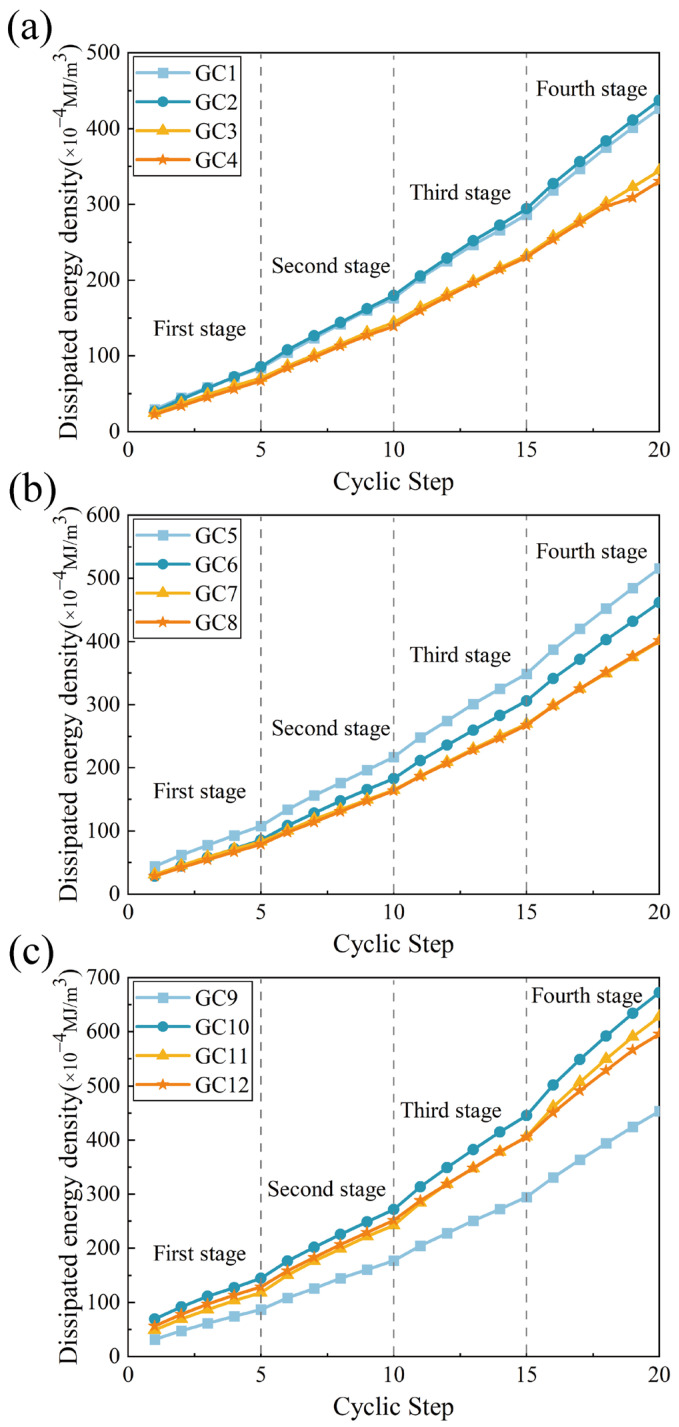
Dissipative energy density curves. (**a**) 20% gangue content group. (**b**) 40% gangue content group. (**c**) 60% gangue content group.

The dissipated energy density curve of the specimen is depicted in Figure 8. In the gangue concrete group with a 20% substitution rate of alternative components, when the polypropylene fiber mixing amount is 0.2% and 0.3%, the difference between the dissipated energies is not significant. However, compared with the fiber-free control group, the dissipated energy is notably reduced, with reductions of 19.19% and 22.53%, respectively. When the polypropylene fiber mixing amount is 0.1%, the dissipated energy is slightly enhanced compared with the fiber-free control group, with an improvement of 2.56%. This indicates that at lower gangue dosages, the lower fiber dosage is insufficient to enhance the connectivity between microscopic particles. Consequently, unevenly distributed micropores form inside the specimen, resulting in higher dissipation energy in the GC2 specimen compared to the GC1 specimen. In the gangue concrete group with a 40% replacement component, the 0.2% and 0.3% polypropylene fiber admixtures exhibited the same pattern as the specimen group with a 20% replacement component. Both groups significantly reduced the dissipation energy of the specimens, with the difference between the two groups being minimal. Their reductions were 22.41% and 22.13%, respectively. At 0.1% polypropylene fiber doping, the dissipation energy of the specimens began to decrease compared to that of the no-fiber control group. However, the difference between the dissipation energies was still not significant, with a reduction of only 10.48%. In the gangue concrete group with a replacement fraction of 60%, all fiber content groups exhibited an increase in dissipation energy compared to the no-fiber control group. This increase was attributed to the overall performance decrease in the concrete, while the addition of fibers enabled the specimens to withstand greater deformation before destruction, thereby exhibiting greater dissipated energy. The improvements in dissipated energy for the three additive groups were 48.35%, 38.37%, and 31.44%, respectively.

At equal fiber content, the various substitution fractions also exhibited a certain pattern. Compared to the 20% gangue concrete component, with a 40% gangue concrete content as the alternative component, each fiber group exhibited a greater effectiveness in reducing the dissipated energy, with reduction amplitudes of 3.50%, 20.01%, and 7.04%, respectively. However, in the group with a 60% gangue concrete content as the alternative component, the addition of fibers did not significantly reduce the dissipated energy. The evolution pattern of energy dissipation does not precisely mirror the strength pattern observed in the cyclic experiment, indicating that there is also a certain amount of energy release in the specimen during the crushing and destruction stage.

### 3.5. Residual Strain

**Figure 9:**
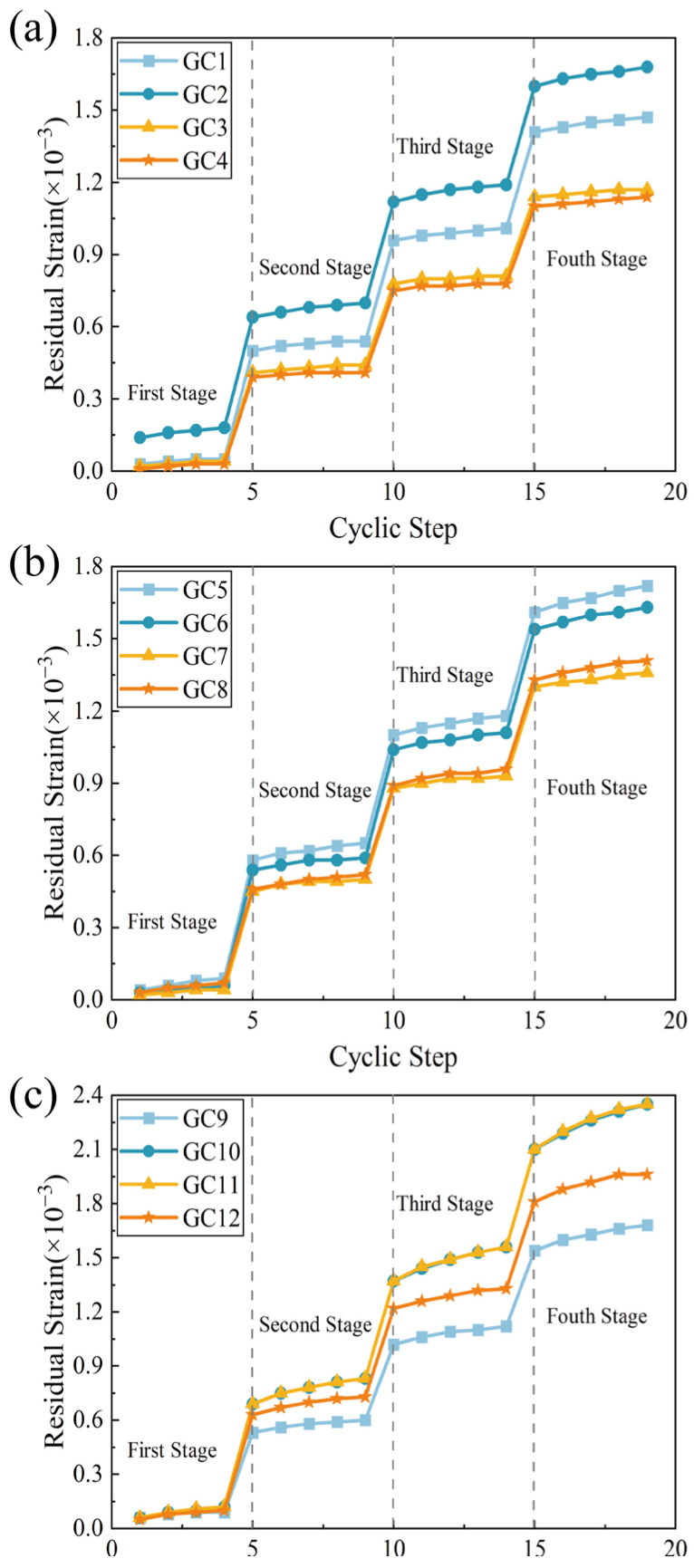
Residual strain curves. (**a**) 20% gangue content group. (**b**) 40% gangue content group. (**c**) 60% gangue content group.

Residual strain serves as a critical indicator of the strain recovery capability of the structure, effectively reflecting the structural resilience against disturbances. The smaller the residual strain, the greater the specimen’s recovery. Figure 9 depicts the variation in residual strain in the specimen for different polypropylene fiber doping levels over a period of 20 cycles.

When the stress amplitude increases, the residual strain of the specimen will undergo an abrupt change, indicating that stress is the primary cause of specimen damage. However, within the same stress amplitude cycle, the damage rate of the specimen gradually decreases with an increase in the number of cycles, indicating an enhancement in the internal densification of the specimen.

The cumulative residual strain values of specimens in groups GC1 and GC5, without added fibers, were notably higher compared to those of specimens in the PPF-added group at 20% and 40% of gangue admixture. This finding substantiates that repetitive stress loading in the absence of PPFs induced greater internal damage to the gangue concrete. This further illustrates that the incorporation of polypropylene fibers effectively mitigates stress concentration within the specimen, enhancing its resistance to disturbances and its ability to recover from strain. While the residual strain value decreased with the increase in fiber content when gangue doping was increased to 60%, the effect of PPFs on reducing the residual strain remained present. At the same time, the cumulative residual strain exhibited a higher overall value compared to the groups with 20% and 40% gangue admixture. This is attributable to the lower crushing value of gangue compared to ordinary crushed stone when subjected to the same repetitive load, rendering concrete with a high gangue content more susceptible to irreversible deformation.

### 3.6. Damage Factors

**Figure 10:**
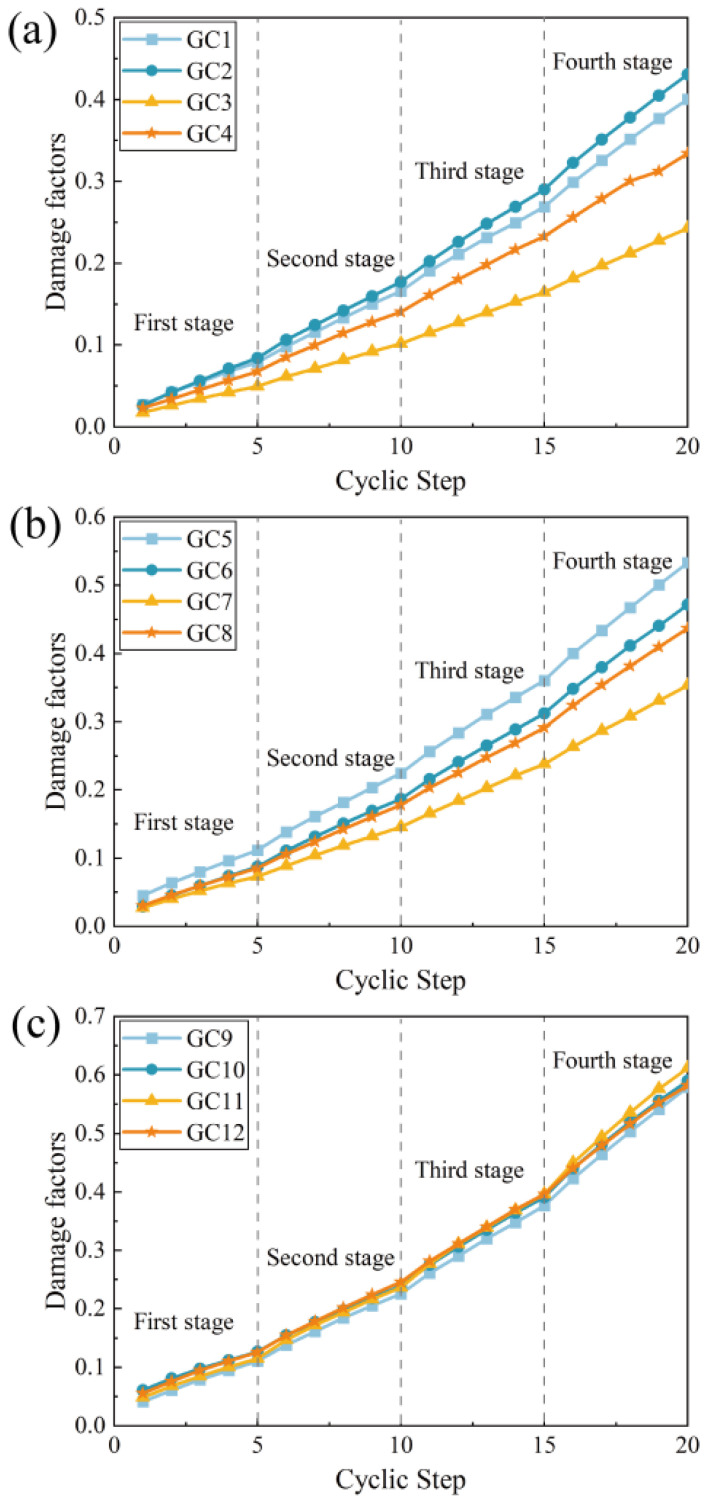
Damage factor curves. (**a**) 20% gangue content group. (**b**) 40% gangue content group. (**c**) 60% gangue content group.

To further investigate the damage characteristics of concrete specimens with different gangue replacement fractions and polypropylene fiber contents, the damage factors were calculated for each cycle, derived from the dissipated energy, as illustrated in Figure 10. The greater the damage factor, the higher the degree of damage the concrete specimen had suffered. The damage factors were calculated using the following formula:

$$D_{i}=\sum_{i=1}^{n}\frac{U_{i}^{d}}{U^{d}}=\sum_{i=1}^{n}\frac{\int \sigma_{i}\varepsilon_{i}d\varepsilon_{i}}{\sum_{i=1}^{n}\int \sigma_{i}\varepsilon_{i}d\varepsilon_{i}}$$

where $D_{i}$ is the damage factor of the *i*th cycle, and $U_{i}^{d}$ is the dissipated energy density of the *i*th cycle, ${\mathrm{MJ}/m}^{3}$.

For each group of samples, the damage factor shows an increasing trend as the number of cycles increases, which shows that repeated loading experiments exert a type of damage behavior on concrete specimens. With the increase in gangue doping from 20% to 60%, the damage factor of the fiber-free specimen increased by 44.5%; the damage factor of the specimen with 0.1% fiber doping increased by 37.2%; the damage factor of the specimen with 0.2% fiber doping increased by 100.55%; and the damage factor of the specimen with 0.3% fiber doping increased by 76.36%. This proves that as the proportion of gangue in concrete increases, the degree of damage suffered by the specimen gradually increases.

When the content of gangue in concrete is 20% and 40%, the damage factor of the fiber-free control group is maintained at a relatively high level. The damage factor of the experimental group containing polypropylene fiber was significantly lower than that of the control group. At 40% gangue content, the cumulative damage factor of gangue fiber concrete with 0.2% fiber content was 33.76% lower than that of the control group. In this case, the addition of polypropylene fibers significantly reduced the damage suffered by gangue concrete when subjected to repeated loads. This can be attributed to the bridging effect of polypropylene fiber on the weak surface of the concrete base. When the concrete is subjected to repeated loads, the fiber bridge transmits stress at both ends of the weak surface and delays the growth of cracks, thereby achieving the effect of reducing the degree of damage.

Furthermore, when the coal gangue content reaches 60% in concrete, there is not a huge gap in the damage factors of each group. At this stage, the coal gangue occupies a large region in the concrete. Due to the low strength, high porosity, and high water absorption of coal gangue itself, the overall strength of the specimens dropped significantly, and the ability of each group of specimens to resist damage under repeated loading was at a relatively low level, which cannot be compensated for via modification by adding polypropylene fiber.

### 3.7. Microscopic Electron Microscope Analysis

**Figure 11:**
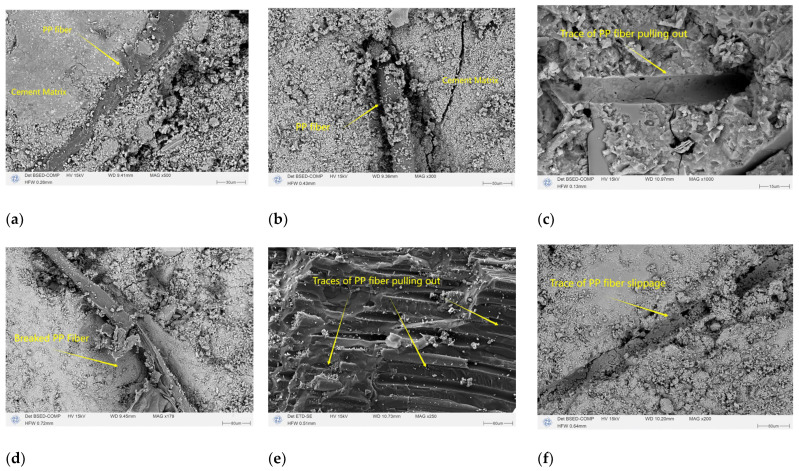
Microscopic morphology of gangue fiber concrete. (**a**,**b**) Bonding effect of PP fibers and cement matrix. (**c**–**f**) Sliding, pulling out, and fracture behavior of PP fibers in cement matrix.

The SEM test results of the gangue fiber concrete are given in Figure 11. From Figure 11a,b, it can be observed that the polypropylene fiber and the cement matrix have a good bonding effect, and the fibers are tightly wrapped and tightly bonded by the cement slurry. Further observations of the slip, fracture, and pull-out behavior of polypropylene fibers are shown in Figure 11c–f. When concrete specimens are subjected to repeated loads, the tight coupling of PP fibers and cement matrix allows PP to withstand some of the stress and to exhibit diverse failure modes. It has been proven from a microscopic perspective that polypropylene fibers in concrete instead of cement matrix can withstand the energy dissipation caused by repeated loading. Polypropylene fibers bear loads in the concrete matrix and transmit stress to avoid stress concentration, thereby improving the fatigue performance of gangue concrete, which is consistent with the conclusions obtained from previous macro-mechanical experiments.

## 4. Conclusions

1. Gangue content inversely affects compressive strength. Fibers enhance strength by crack inhibition, but gangue content remains decisive.
2. Polypropylene fibers enhance gangue concrete strength under cyclic loading, notably at 20% and 40% gangue substitution fractions.
3. Polypropylene fibers reduce dissipated energy in gangue concrete, with varied effects based on content and ratio. Increased stress amplitude causes abrupt residual strain changes, mitigated by polypropylene fibers, enhancing specimen resistance to damage.
4. Repeated loading increases the damage factor in concrete specimens. The higher the gangue content, the greater the damage. Polypropylene fiber can reduce the damage, with a maximum reduction of 33.76%.
5. SEM tests show that polypropylene fibers are tightly bonded to the cement matrix, bearing loads and transmitting stress.

## Figures and Tables

**Table 1:** Gangue concrete mixing ratio.

Group	Gangue Content	Cement/g	Sand/g	Stone/g	Gangue/g	Water/g	Water Reducer/g	PP Fiber Content
GC1	20%	445	641	911	207	178	3.3	0
GC2	20%	445	641	911	207	178	3.3	0.1%
GC3	20%	445	641	911	207	178	3.3	0.2%
GC4	20%	445	641	911	207	178	3.3	0.3%
GC5	40%	445	641	683	414	178	3.3	0
GC6	40%	445	641	683	414	178	3.3	0.1%
GC7	40%	445	641	683	414	178	3.3	0.2%
GC8	40%	445	641	683	414	178	3.3	0.3%
GC9	60%	445	641	456	620	178	3.3	0
GC10	60%	445	641	456	620	178	3.3	0.1%
GC11	60%	445	641	456	620	178	3.3	0.2%
GC12	60%	445	641	456	620	178	3.3	0.3%

## Data Availability

The data that support the findings of this study are available from the corresponding author, [D.W.], upon reasonable request.
